# Antiviral activity of temporin-1CEb analogues against gingival infection with herpes simplex virus type 1

**DOI:** 10.3389/froh.2024.1430077

**Published:** 2024-06-17

**Authors:** Anna Golda, Paulina Kosikowska-Adamus, Marta Wadowska, Ewelina Dobosz, Jan Potempa, Joanna Koziel

**Affiliations:** ^1^ Department of Microbiology, Faculty of Biochemistry, Biophysics and Biotechnology, Jagiellonian University, Krakow, Poland; ^2^ Department of Organic Chemistry, Faculty of Chemistry, University of Gdansk, Gdansk, Poland; ^3^ Department of Oral Immunology and Infectious Diseases, University of Louisville School of Dentistry, University of Louisville, Louisville, KY, United States

**Keywords:** herpes simplex virus, antimicrobial peptides, temporins, heparan sulfate, gingival infection, drug-resistance

## Abstract

**Introduction:**

Oral herpes infections caused by herpes simplex virus type 1 (HSV-1) are one of the most common in the human population. Recently, they have been classified as an increasing problem in immunocompromised patients and those suffering from chronic inflammation of the oral mucosa and gums. Treatment mainly involves nucleoside analogues, such as acyclovir and its derivatives, which reduce virus replication and shedding. As drug-resistant strains of herpes emerge rapidly, there is a need for the development of novel anti-herpes agents. The aim of the study was to design an antiviral peptide, based on natural compounds, non-toxic to the host, and efficient against drug-resistant HSV-1. Here, we designed a lysine-rich derivative of amphibian temporin-1CEb conjugated to peptides penetrating the host cell membrane and examined their activity against HSV-1 infection of oral mucosa.

**Methods:**

We assessed the antiviral efficiency of the tested compound in simple 2D cell models (VeroE6 and TIGKs cells) and a 3D organotypic model of human gingiva (OTG) using titration assay, qPCR, and confocal imaging. To identify the molecular mechanism of antiviral activity, we applied the Azure A metachromatic test, and attachment assays techniques. Toxicity of the conjugates was examined using XTT and LDH assays.

**Results:**

Our results showed that temporin-1CEb analogues significantly reduce viral replication in oral mucosa. The mechanism of peptide analogues is based on the interaction with heparan sulfate, leading to the reduce attachment of HSV-1 to the cell membrane. Moreover, temporin-1CEb conjugates effectively penetrate the gingival tissue being effective against acyclovir-resistant strains. Collectively, we showed that temporin-1CEb can be regarded as a novel, naturally derived antiviral compound for HSV-1 treatment.

## 1 Introduction

Herpes simplex virus type 1 (HSV-1) is a double-stranded human DNA virus belonging to the *Herpesviridae* family. Infections with HSV-1 are widespread worldwide, and according to the WHO, 3.7 billion people (constituting 67% of the global population) under the age of 50 are seropositive for HSV-1 (1). HSV-1 infection is incurable and persists throughout the host's life, often in a latent form. Such an infection may be asymptomatic or manifest as mucocutaneous infections (oral and labial herpes) and genital herpes (2). Importantly, the significant risk for the transmission of HSV-1 occurs during phases of unrecognized or asymptomatic shedding (3, 4). Occasionally, HSV-1 infection can lead to more severe conditions such as herpetic encephalitis (5, 6) which has a mortality rate of about 70% in the absence of treatment (7). Moreover, the virus is a source of rare infections in newborns (8) which, if left untreated, pose a serious condition with an estimated 60% fatality rate (9). Furthermore, ocular herpes simplex infection is a leading cause of corneal blindness in developed countries (10, 11). Recently, HSV-1 infection pays a lot of attention as there are epidemiological and clinical studies suggesting its comorbidity with neurodegenerative conditions, including Alzheimer's disease (12–20).

Currently, the first-line medications used in the treatment of herpes infections are acyclovir, valacyclovir, and famciclovir (2). These guanosine analogs lead to the inhibition of viral polymerase and viral DNA synthesis. Acyclovir-based therapies and their derivatives limit the duration and severity of primary infection symptoms; however, they do not eliminate the virus in its latent state (2, 21). Additionally, the emergence of isolates resistant to these drugs is increasingly observed, especially in immunocompromised patients, such as those with active HIV infection or following bone marrow transplants (21). Second-line therapies, cidofovir and foscarnet, are medications approved for the treatment of acyclovir-resistant HSV in immunocompromised hosts; however, their use is limited by significant cytotoxicity (2, 21). Furthermore, there are no approved HSV-1 vaccines available for either prophylactic or therapeutic use, though there are different candidates in the pre-clinical and clinical phases of study (21, 22). In this context, there is a need for the development of novel anti-herpes agents.

Antimicrobial peptides (AMPs), alternatively referred to as host defense peptides (HDPs), are naturally occurring molecules expressed in humans, animals, and plants (23, 24). AMPs are part of the host's innate immune response and exhibit antimicrobial activity against a broad range of microorganisms. They do not induce the development of resistance; therefore they constitute a promising alternative to conventional antimicrobial drugs (25, 26). Among peptides with broad therapeutic applications are temporins isolated from the granular glands of the skin of frogs belonging to the genus *Rana* (27). Temporins are short (10–14 amino acids), hydrophobic, and C-terminally amidated AMPs with more than 100 different isoforms altogether (28). Members of the temporin family exhibit strong antibacterial activity against both Gram-positive and Gram-negative bacteria, as well as fungi (28). In recent years, several reports have highlighted their high efficiency against HSV-1, including temporin B, L, and G. Research findings suggest that temporin B acts by disrupting the lipid envelope of HSV-1 and partially inhibits various steps of the viral replication cycle, including adhesion, cell entry, and subsequent replication stages (29). Likewise, temporin L inhibits herpesvirus infection mainly through direct interaction with virion (30). Moreover, temporin G exhibits antiviral activity by acting either during the earliest of viral life cycle or directly affecting viral particles (31). These data indicate the significant potential of temporins against viral pathogens, particularly HSV-1.

The aim of the study was to design an antiviral peptide based on natural compound, non-toxic to the host and efficient against drug-resistant HSV-1. Here, we have prepared a set of conjugates of temporin-1CEb analogues (LK6, DK5) with peptides capable of penetrating intracellularly (CCP1/CCP2) and/or promoting tissue regeneration (DAL). In the presented study, we examined their antiviral efficiency and identified their molecular mechanism of activity, finding CPP1-PEG-LK6 to be the most potent against HSV-1 and non-toxic to the host. Moreover, due to its effectiveness against drug-resistant HSV-1, we propose it as a novel promising agent against mucosal infections.

## 2 Material and methods

### 2.1 Peptides synthesis and purification

All compounds were synthesized manually under the standard conditions using the solid-phase method applying to the 9-fluorenyl-9methoxycarbonyl (Fmoc)/tert-butyl (tBu) strategy. TentaGel S RAM (Rapp Polymere GmbH) with a substitution degree of 0.24 mmol/g was utilized as the solid support to ensure an amidated C-terminal end in the final compounds. The peptide chain was elongated using Fmoc-protected amino acids (3 equiv), combined with HOBt (N-hydroxybenzotriazole)/HBTU [N,N,N’,N’-Tetramethyl-O-(1H-benzotriazol-1-yl) uronium hexafluorophosphate] (3 equiv) as coupling reagents, and DIPEA (N,N-Diisopropylethylamine) (6 equiv) as a non-nucleophilic organic base to facilitate the binding of the subsequent residues. The completeness of each coupling step was monitored using both Kaiser and chloranil tests. After each coupling, the Fmoc protection was removed with 20% piperidine in dimethylformamide (DMF). PEG linker (Fmoc-O2Oc-OH, Iris Biotech, Germany) was introduced into the peptide sequence under the same reaction conditions. Upon completion of the synthesis, the N-terminal F-moc protection was removed and peptides were cleaved from the resin using a mixture of TFA/phenol/triisopropylsilane/H2O (88:5:2:5, v/v).

The synthesized compounds were then purified by reverse-phase high-performance liquid chromatography (RP-HPLC) on a Waters system (Phenomenex Jupiter 4 μ Proteo 90 Å column, 250 × 10 mm). A linear gradient from 10% to 80% of solvent B over 60 min (Solvent A: 0.1% TFA in water; Solvent B: 80% acetonitrile in Solvent A) was employed with a flow rate 5 ml/min. The homogeneity of the final peptide fractions was analyzed using a Shimadzu HPLC System (Shimadzu Europe GmbH, Duisburg, Germany) equipped with a Phenomenex Jupiter 4 μ Proteo 90 Å column, 250 × 4.60 mm. The mass spectra of the synthesized peptides were recorded using a Biflex III MALDI-TOF mass spectrometry (Bruker, Mannheim, Germany) with *α*-cyano-4-hydroxy-cinnamic acid (CCA) or 2,5-dihydroxybenzoic acid (DHB) used as the matrix.

### 2.2 Herpes simplex virus type 1 (HSV-1)

HSV-1 strain F was acquired from American Type Culture Collection (VR 733). Clinical isolates of HSV-1 (294.1, 615.9, C08R, C10B) were kindly provided by Donald M. Coen from Department of Biological Chemistry and Molecular Pharmacology, Harvard Medical School, Boston. HSV-1 strains were propagated and titrated on Vero cells.

### 2.3 Virus preparation and titration

HSV-1 stocks were generated by infecting Vero E6 cells. Cells were lysed at 48 h p.i. by two freeze-thaw cycles. The virus containing fluid was aliquoted and stored at −80°C. A control Vero E6 cell lysate from mock-infected cells was prepared in the same manner as the virus stock. Virus yield was assessed by virus titration on fully confluent Vero E6 cells in 96-well plates, according to the method of Reed & Muench (32). Plates were incubated at 37°C for 48 h and the occurrence of a cytopathic effect was scored using an inverted microscope, and TCID_50_ (50% tissue culture infection dose) was calculated.

### 2.4 Virus inhibition assay

Vero E6 or TIGKs cells were seeded in 12-well plates and cultured for 48 h. Next, the medium was removed and replaced with a fresh medium supplemented with DAL-PEG-LK6 and CPP1-PEG-LK6 (15 μg/ml). After 30 min of incubation at 37°C, cells were infected with HSV-1 at TCID_50_ 400 /ml or treated with mock. After 2 h of infection at 37°C, unbound viral particles were removed by washing with PBS, and culture media supplemented with peptide conjugates were applied. Following 48 h of incubation, media were collected, and the quantity of HSV-1 DNA copies was assessed using titration and qPCR.

### 2.5 Virion integrity assay

HSV-1 samples were incubated with DAL-PEG-LK6 and CPP1-PEG-LK6 (15 μg/ml) for 1 h at room temperature on a shaker. Additionally, Triton X-100 was used as a positive control. Next, the mixtures were diluted to decrease tested molecules concentrations (0.65 ng/ml) and were used to infect TIGKs monolayers. After 48 h of incubation, supernatants were collected, and the quantity of HSV-1 DNA copies was determined using qPCR.

### 2.6 Attachment assay

TIGKs cells were seeded in 12-well plates and cultured for 48 h. Next, keratinocytes were infected with HSV-1 (TCID_50_ 400 /ml) or treated with mock in the presence of DAL-PEG-LK6 or CPP1-PEG-LK6 (15 μg/ml) for 2 h at 4°C. Following three washes with PBS to remove both the unattached virus and peptide, cells were incubated for 48 h at 37°C. Next, samples were collected, and viral yield was determined using titration and qPCR.

### 2.7 Cell lines

Vero E6 cells (ATCC CRL-1586) were maintained in DMEM supplemented with 3% heat-inactivated fetal bovine serum (FBS, Life Technologies), penicillin (100 U/ml), and streptomycin (100 μg/ml). Telomerase-immortalized gingival keratinocytes (TIGKs) were generated from primary gingival epithelial cells (GECs) and kindly provided by R. J. Lamont (33). Cells were cultivated in KBM-Gold keratinocyte basal medium supplemented with Single Quots (Lonza). Immortalized Human Gingival Fibroblasts-hTERT (T0026, Applied Biological Materials) were cultivated on collagen-coated bottles in PriGrow III medium (Applied Biological Materials) supplemented with 10% heat-inactivated FBS. Cell cultures were maintained in the atmosphere containing 5% CO_2_ at 37°C, and when they reached 80%–90% confluency, cells were seeded for experiments.

### 2.8 3D gingival model

Protocol was described previously (34). Briefly, to prepare 3D cultures, 150,000 gingival fibroblasts suspended in 10% FBS in DMEM were mixed with 2.4 ml of ice-cold Matrigel® (Corning) (final concentration 7 mg/ml) and placed in the middle of 12-well cell culture inserts (Greiner). Three days later, 1 × 10^6^ TIGK cells suspended in EPM1 were added to the top of the insert. On the ninth day after the start of the procedure, the old culture medium was removed and EPM2 was added to the bottom of the insert. The epithelial layer on the top was exposed to the air, which promotes epithelial differentiation and then stratification. About 8–10 days after air-liquid interphase, 3D cultures of gingiva were used for further experiments.

The apical surface of OTG model was washed three times with PBS and then inoculated with 100 μl of viral stock (TCID_50_ 400 /ml) in the presence of DAL-PEG-LK6 or CPP1-PEG-LK6 (15 μg/ml) for 2 h at 4°C. Following incubation, the unbound HSV-1 was removed by washing with PBS for 10 min at 37°C, and the OTG cultures were maintained at an air-liquid interface for the rest of the experiment. To determine viral yield, 120 μl of PBS was applied to the apical surface of the OTG, and harvested for DNA isolation after 10 min of incubation at 37°C.

### 2.9 Histological staining of tissue

Briefly, cell inserts were fixed in 10% formaldehyde at 4°C for 1 h. After embedding in paraffin, 3D cultures were cut into 5 μm sections and stained with H&E solution. For immunohistochemical analysis, slides were incubated for 1 h at room temperature in blocking buffer (5% normal goat serum, 0.1% saponin in PBS) and stained with primary antibodies anti-HSV-1 (1:100, 1% normal goat serum, 0.1% saponin in PBS; Abcam) and secondary antibodies: goat anti-rabbit conjugated with Alexa Fluor 488 (1:2000; Cell Signaling). Finally, cell nuclei were stained with ProLong Antifade Mounting Medium with DAPI. Slides were visualized with EVOS FL Cell Imaging System (Thermo Fisher Scientific).

### 2.10 Cell viability tests

The viability of Vero E6 and TIGKs cells was examined by the XTT Cell Viability Assay kit (Biological Industries, USA). Briefly, cells were treated with indicated concentrations of the peptide conjugates for 48 h at 37°C. Next, the fresh medium was added to the cells and 50 μl of the activated XTT reagent [2,3-bis(2-methoxy-4-nitro-5-sulphenyl)-2H-tetrazolium-5-carboxanilide] was added and further incubated for 2 h at 37°C. Absorbance was measured at a wavelength of 480 nm using FlexStation 3 microplate reader (Molecular Device, Wokingham, UK). The results were expressed normalized against a control of untreated cells (100% viability). To assess the effect of peptide conjugate on the integrity of the plasma membrane, the LDH release assay was performed using the CytoTox96 nonradioactive cytotoxicity assay kit (Promega, Madison, WI, USA) according to the manufacturer's instructions. Cytotoxicity was calculated with the formula: % cytotoxicity = 100 × (experimental LDH release/ maximum LDH release), where maximum LDH release is after lysis solution addition (Triton X-100). Relative amounts of LDH release were measured (absorbance at 490 nm) using FlexStation 3 microplate reader (Molecular Device, Wokingham, UK). All assays were performed in triplicate.

### 2.11 Confocal scanner microscopy

TIGKs cells were seeded on coverslips for 48 h in KBM-Gold medium supplemented with Single Quots. Next, cells were infected with HSV-1 in the presence of DAL-PEG-LK6 and CPP1-PEG-LK6 at 4°C (adsorption/attachment assay). After 24 h p.i., cells were washed with PBS and fixed with 3.7% formaldehyde for 30 min. Afterwards, cells were incubated with blocking buffer and stained with primary antibodies (1:100; rabbit anti-HSV-1, Abcam) for 1 h at room temperature. The cells were then incubated with secondary antibodies (1:2,000; goat anti rabbit conjugated with Alexa Fluor 488, Cell Signaling) and with Alexa Fluor 633 Phalloidin. Finally, the coverslips were placed on glass slide with ProLong Antifade Mounting Medium with DAPI and visualized using a confocal laser scanning microscope (LSM 880; Zeiss).

To examine the colocalization of HS on the cell surface with tested peptide conjugate we treated TIGKs cells with CFS stained CPP1-PEG-LK6 (15 μg/ml) for 30 min at 4°C. Then cells were washed with cold PBS and fixed with 3.7% formaldehyde for 30 min. Afterwards, TIGKs cells were stained with primary antibodies (1:100; rat anti-HS, Abcam) for 2 h at room temperature. The cells were then incubated with secondary antibodies (1:1,000; goat anti-rat conjugated with Alexa Fluor 568, Cell Signaling) and with Alexa Fluor 633 Phalloidin. Next, the coverslips were placed on glass slide with ProLong Antifade Mounting Medium with DAPI and visualized using a confocal laser scanning microscope (LSM 880; Zeiss).

### 2.12 Virus detection by qPCR

Viral DNA was isolated from the conditioned media or the apical washes of inserts (OTG model) using Viral DNA/RNA Isolation Kit (A&A Biotechnology). The virus yield assessed by quantitative real-time PCR (qPCR). The reaction was carried out in a CFX96 Touch Real-Time PCR Detection System (Bio-Rad), in a 10 μl reaction mixture consisting of TaqMan^TM^ Fast Advanced Master Mix, TaqMan^TM^ Microbe Detection assay against HSV-1 (Vi04230116_s1) and 1 μl of viral DNA. The temperature profile was 10 s at 95°C, followed by 40 cycles of 3 s at 95°C and 20 s at 60°C. DNA quantification standards were prepared. In summary, a fragment of viral DNA was amplified using the primers listed above and cloned into the pTZ57R/T plasmid using the InsTAclone PCR cloning kit. The plasmid was propagated in *E. coli*, purified with the GeneJet Plasmid Miniprep Kit, and digested with the EcoR1 restriction enzyme. The number of copies per mL was estimated after the concentration of linearized DNA was assessed spectrophotometrically.

### 2.13 Azure A metachromatic assay

The possible interaction of peptide conjugate with HS was investigated by incubation the mixtures containing a constant concentration of HS (0.2 mg/ml, Sigma Aldrich) and solutions of CPP1-PEG-LK6 at different concentrations (1–300 μg/ml). Next, the mixtures were shaken for 15 min at room temperature. Finally, to 100 μl of the sample 100 μl of Azure A solution (23 μg/ml, Sigma Aldrich) was added and the absorbance was measured (400–800 nm) using FlexStation 3 microplate reader (Molecular Device, Wokingham, UK). Azure A, a cationic dye when binding to heparin and/or HS changes its adsorption spectrum (35).

### 2.14 Western blot analysis

Protein extracts were prepared using lysis buffer (0.25% Na-deoxycholate, 0.5% Nonidet P-40, 0.05% SDS, protease inhibitor mixture, 2.5 mM EDTA in PBS), and the protein concentration was assessed using Pierce BCA (Thermo Fisher) protein assay kit. Equal amounts of cell extracts were separated on 10% SDS-PAGE gel and electrotransferred onto polyvinylidene difluoride membranes for gB and β-actin in transfer buffer (25 mM Tris, 0.2 M glycine, and 20% ethanol) for 90 min at 100 V. Nonspecific binding sites were blocked for 90 min in 5% skimmed milk prepared in TBST buffer (20 mM Tris, pH 7.5, 0.5 M NaCl, 0.05% Tween 20). Membranes were further incubated overnight at 4°C with primary antibodies: mouse anti-HSV-1 + HSV-2 gB (1:5000; abcam) and mouse anti-β-actin (1:4,000; BD Biosciences). The following day, membranes were washed three times with TBST buffer and incubated with HRP-conjugated secondary antibodies for 60 min at 37°C: goat anti-mouse IgG (1:20,000; β-actin; BD Bioscience) or sheep anti-mouse IgG (1:50,000; gB; Sigma Aldrich). Next, blots were washed five times for 5 min in TBST buffer, developed using Luminata Cresecendo Substarte (Merck) and exposed to AGFA medical x-ray film. Densitometry analysis was performed using ImageJ 1.52q software.

### 2.15 Statistical analysis

Data were expressed as means ± SD. Statistical significance was determined using unpaired *t*-test, one-way ANOVA, and/or two-way ANOVA for direct comparisons between single groups. A *p*-value <0.05 was considered statistically significant. We used GrapPad Prism 10 software for the analyses. The concentration causing a 50% reduction in herpes infection (IC_50_) and compound concentration required to reduce the cell viability by 50% (CC_50_) was determined by regression analysis using GraphPad Prism 10 software by fitting a variable slope-sigmoidal dose–response curve.

## 3 Results

### 3.1 Antiviral activity of peptide conjugates with temporin-1CEb analogues

**Figure 1:**
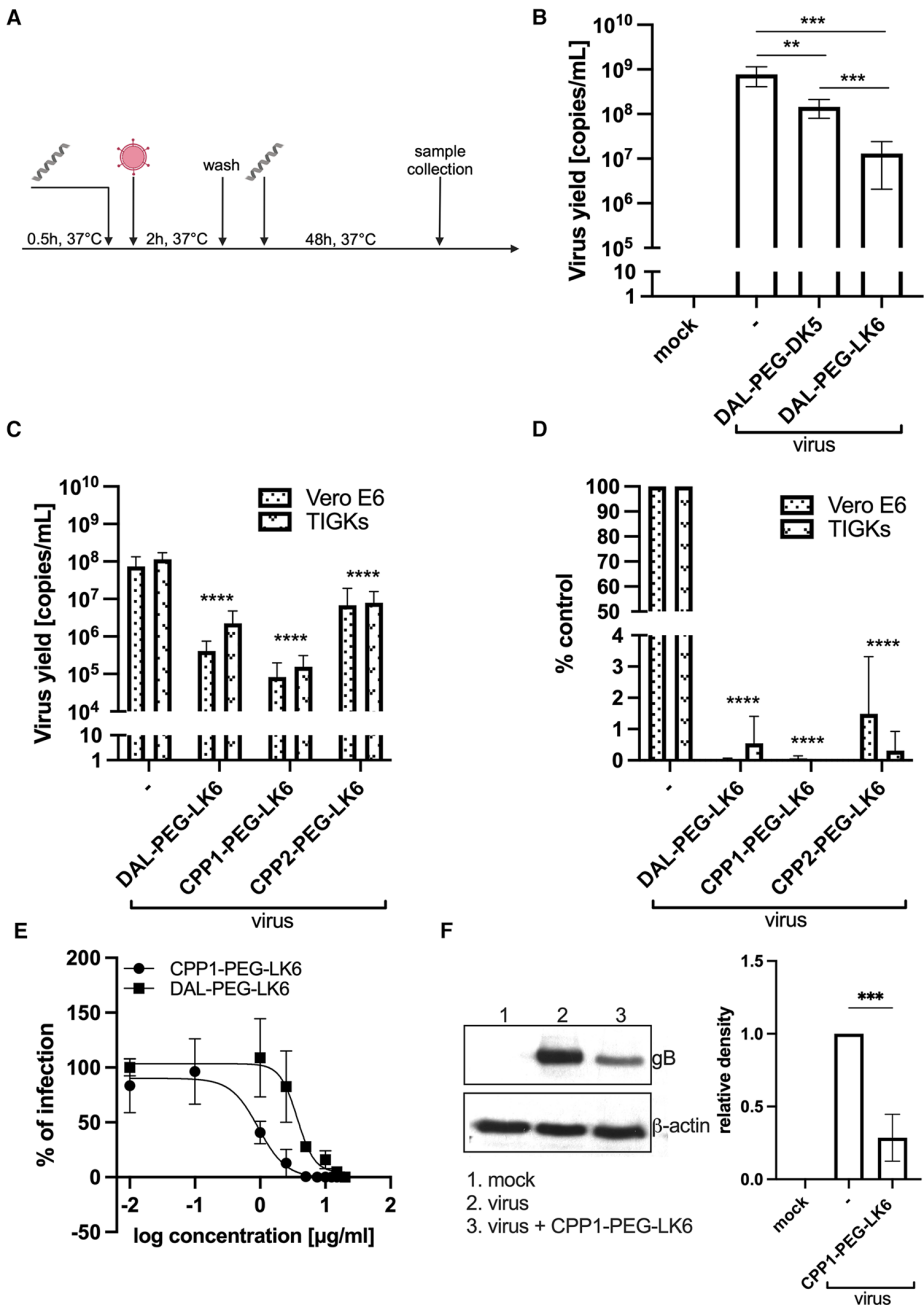
Antiviral activity of temporin-1CEb analogues. (**A**) Schematic representation of the experiment. (**B**) DAL-PEG-DK5 and DAL-PEG-LK6 (20 μg/ml) were present before, during, and after HSV-1 infection. Vero E6 cell culture supernatants were collected 48 h p.i., and the amount of viral DNA was determined by qPCR. Results are presented as an average ± SD of three independent experiments in duplicates. **P < 0.01, ***P < 0.001; unpaired *t*-test. Next, the assay was carried out in Vero E6 (DAL-PEG-LK6 and CPP1-PEG-LK6 25 μg/ml; CPP2-PEG-LK6 10 μg/ml) and TIGKs cells (all peptide conjugates 15 μg/ml). Viral yield was quantified by qPCR (**C**) or titration (**D**) For qPCR, data are shown as virus yield (copies of viral genome/ml) and for virus titration, obtained results were normalized to the control sample of peptide conjugate-untreated cells. Results are presented as an average ± SD of four independent experiments in duplicates. ***P < 0.001, ****P < 0.0001; one-way and 2wayANOVA. (**E**) Evaluation and comparison of the antiviral activity of DAL-PEG-LK6 and CPP1-PEG-LK6. TIGKs cells were pre-treated with DAL-PEG-LK6 and CPP1-PEG-LK6, infected with HSV-1 in the presence of tested conjugates and cultivated in their presence at the indicated concentrations. Inhibition of the herpes infection was evaluated using qPCR. The concentration causing a 50% reduction in HSV-1 infection (IC_50_) of the compounds was calculated by regression analysis. Data are shown as the mean ± SEM of two independent experiments in duplicates. (**F**) CPP1-PEG-LK6 significantly suppressed the expression level of gB. CPP1-PEG-LK6 (15 μg/ml) was present before, during and after viral infection. After 48 h p.i., cell lysates were harvested, and the expression level of gB was detected by Western blot. The optical density of the gB bands was normalized against β-actin. The normalized density of the virus-infected sample was arbitrarily set to 1. ***P < 0.00; unpaired t test.

Recently, we showed that temporin-1CEb analogues are efficient compounds against infection with intracellular bacteria (36). Therefore, our objective was to examine whether they could also exert antiviral activity. For that purpose, we applied recently described temporin-1CEb analogues (DK5 and LK6), synthesized with several L-Lys and D-Lys substitutions, which increased the net charge of DK5 and LK6 from +1 (temporin-1 CEb) to +6 and +7, respectively (37). DK5 and LK6 were characterized as *α*-helical cationic peptides with a broad range of antimicrobial properties and low cytotoxicity against eukaryotic cells (37). These peptides were conjugated with dalargin (DAL), a synthetic Leu-enkephalin analogue that stimulates regeneration of injured tissues, reduces oxidative stress, and pain (38). The peptides were covalently bound to DK5 and LK6 by means of a PEG (O2Oc) linker (0-amino-3,6-dioxaoctanoic acid) (39). Both conjugates (DAL-PEG-DK5 and DAL-PEG-LK6) (39) were used to determine the antiviral activity against HSV-1 using a virus replication assay. For this purpose, we applied an *in vitro* model of Vero E6 cells, commonly used to study HSV-1 (40, 41). These cells were pre-stimulated with the tested compounds (Figure 1A), at non-toxic doses 20 μg/ml and 10 μg/ml (36) (Supplementary Figure S1), before infection with HSV-1. After infection, cells were cultivated for 48 h in the presence of tested conjugates (Figure 1A). The replication of HSV-1 was examined up to 48 h post-infection using quantitative polymerase chain reaction (qPCR). The obtained results (Figure 1B) indicate decreased HSV-1 replication in the presence of both peptide conjugates, albeit the stronger inhibitory effect was observed for DAL-PEG-LK6. Then, we focused on the LK6 temporin analogue and designed peptide conjugates substituting dalargin (DAL) with cell-penetrating peptides CPP1 or CCP2 (Table 1). CCP1 refers to a hydrophobic peptide, which constitutes a C-terminal fragment of the synthetic C105Y peptide, derived from the sequence of α1-antitrypsin (42), while CCP2 refers to a cationic peptide, a fragment of the HIV-1 TAT*49–57* protein (43). Briefly, we examined the toxicity of the novel molecules using XTT and LDH assay, finding that they do not affect the viability of tested cells at concentration below 15 μg/ml (Supplementary Figures S1A–F). Then, we assessed the influence of the non-toxic dose of peptides against virus replication using qPCR and titration methods, applying Vero E6 cells, but also TIGK cells. We found the strongest antiviral activity of CPP1-PEG-LK6, manifested by a significant reduction (3 log) in viral yield in the qPCR test (Figure 1C). On the other hand, DAL-PEG-LK6 and CPP2-PEG-LK6 were less effective in the inhibition of HSV-1 infection: 2 log and 1 log, respectively (Figure 1C). In the case of the titration assay, we observed equally efficient inhibition of virus replication in both cell models regardless of the tested compound (Figure 1D), reaching over 99% (DAL-PEG-LK6 and CPP1-PEG-LK6) and 97% (CPP2-PEG-LK6) reduction. Together, the obtained results indicate that temporin-1CEb analogues are promising candidates against HSV-1. Therefore, for further experiments, we selected DAL-PEG-LK6 and CPP1-PEG-LK6 conjugates. To quantify the antiviral efficiency of tested compounds, we measured the inhibitory concentration 50% (IC_50_) of HSV-1 replication inhibition in TIGKs cells using qPCR (Figure 1E; Table 2). We demonstrated that DAL-PEG-LK6 and CPP1-PEG-LK6 inhibit HSV-1 in a dose-dependent manner. CPP1-PEG-LK6 exhibited higher potential, with an IC_50_ value of 0.942 μg/ml and a higher SI compared to DAL-PEG-LK6 (Table 2). To confirm the above, we showed that CPP1-PEG-LK6 significantly suppressed the expression level of gB (Figure 1F).

**Table 1:** Primary structures of the peptide conjugates with their basic physicochemical characteristic.

Compound	Sequence^a^	MW (calc)	MW (found)	Net charge (at pH 7)	GRAVY index^b^	R*_t_*
DAL-PEG-DK5	Y*a*GFLR-O2Oc-IKKILS*k*IKKLL-NH_2_	2,276.89	2,276.44	+7	0.378	22.550
DAL-PEG-LK6	Y*a*GFLR-O2Oc-IKKILSKIKKLLK-NH_2_	2,405.06	2,404.58	+8	0.558	23.958
CPP1-PEG-LK6	PFVYLI-O2Oc-IKKILSKIKKLLK-NH_2_	2,430.15	2,428.91	+7	0.689	25.247
CPP2-PEG-LK6	RKKRRQRRR-O2Oc-IKKILSKIKKLLK-NH_2_	3,018.83	3,018.32	+15	−1.709	20.094

^a^ Amino acids residues written in lowercase italics refer to D-amino acids, respectively, *a*—D-Ala, *k*—D-Lys.

^b^ Grand average hydropathy (GRAVY) index was calculated using the Expasy ProtParam tool (https://web.expasy.org/protparam/). The calculations considered the amino acid residues only, excluding the PEG linker.

**Table 2:** Peptide conjugates antiviral activity against HSV-1.

Compound	CC_50_^a^ [μg/ml]	IC_50_^a^ [μg/ml]	SI^b^
DAL-PEG-LK6	33.80	3.699	9.14
CPP1-PEG-LK6	23.40	0.942	24.84

^a^ The IC_50_ (inhibitory concentration that reduced viral replication by 50%) and CC_50_ (50% cytotoxic concentration) are expressed as the mean [μg/ml] of two independent experiments.

^b^ SI, selectivity index, determined by the ratio of CC_50_ to IC_50_.

### 3.2 Temporin-1CEb peptide conjugates inhibit adhesion and replication of HSV-1

**Figure 2:**
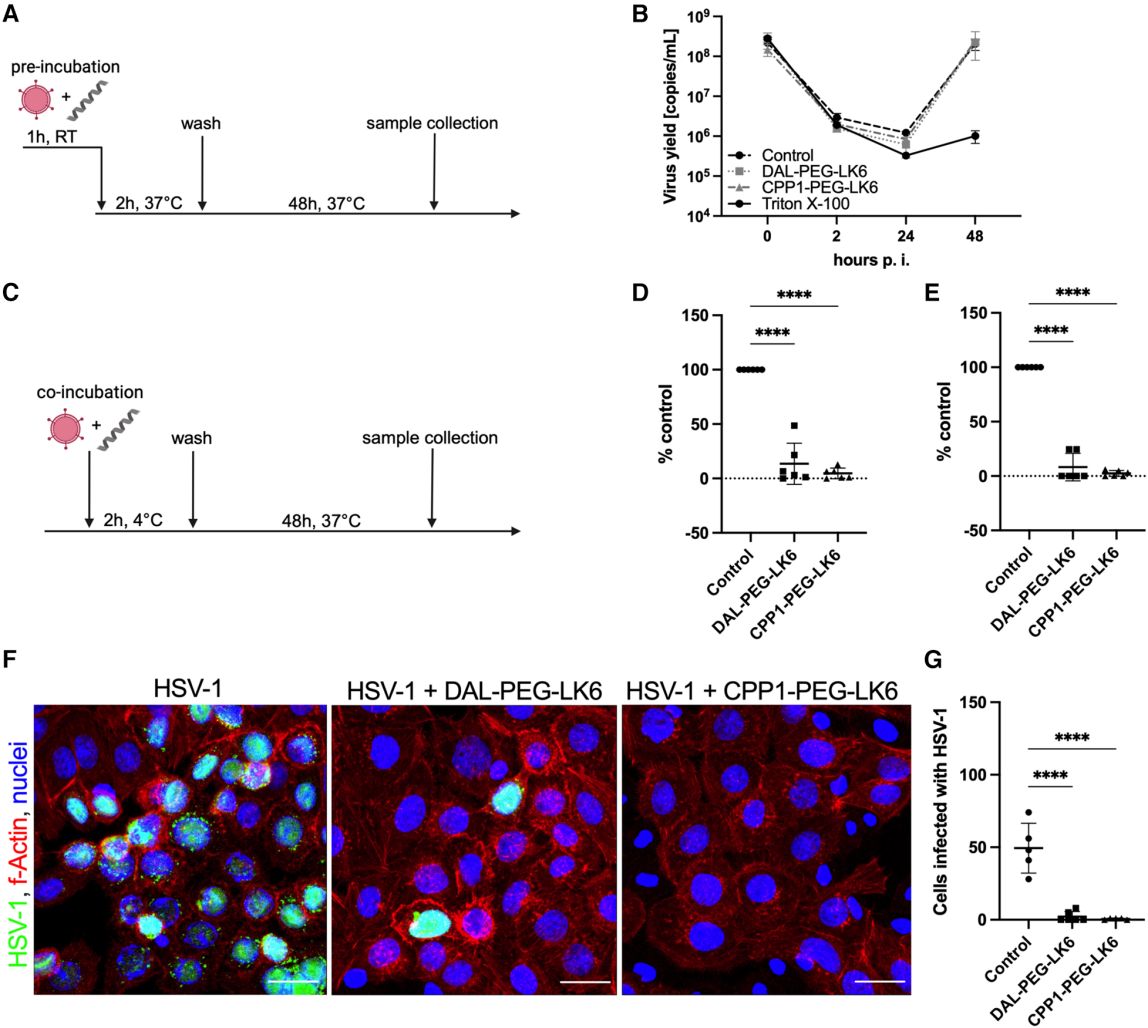
Inhibition of HSV-1 adhesion to the host cell by temporin-1CEb analogues. (**A**) Schematic representation of the virus inhibition assay where the direct effect of tested compound on virion integrity was evaluated. RT, room temperature. (**B**) Firstly, virus particles were pre-treated with DAL-PEG-LK6 and CPP1-PEG-LK6 (15 μg/ml) or Triton X-100 as a positive control. Next, TIGKs cells were infected with such virions and viral yield was assessed up to 48 h p.i. by qPCR. (**C**) Schematic representation of adsorption assay where peptides conjugates were present only during the infection at 4°C. The influence of DAL-PEG-LK6 and CPP1-PEG-LK6 (15 μg/ml) on viral adsorption to epithelial cells was verified. Samples were analyzed by qPCR (**D**) and virus titration (**E**), obtained results were normalized to the control sample of peptide conjugate-untreated cells. Results are presented as an average ± SD of three independent experiments in duplicates. ****P < 0.0001; one-way ANOVA. (**F**) The observation was confirmed by confocal microscopy (virus, green; nuclei, blue; f-Actin, red). Scale bar: 20 μm. (**G**) The number of virus positive cells was quantified using ImageJ 1.52q software.

To examine the molecular mechanism of the antiviral activity of the tested compounds, we applied several models of their distribution (Figures 2A,C). To assess the direct effect of peptides conjugates on virion integrity, HSV-1 particles were pretreated for 1 h at RT with DAL-PEG-LK6, CPP1-PEG-LK6, or Triton X-100 as a positive control before infecting TIGKs cells (Figure 2A). Next, the viral yield was examined up to 48 h post-infection using qPCR. As shown in Figure 2B, both peptides showed no inhibitory effect compared to Triton X-100, suggesting that DAL-PEG-LK6 and CPP1-PEG-LK6 do not affect HSV-1 virion integrity. Then, we explored the effect of DAL-PEG-LK6 and CPP1-PEG-LK6 on HSV-1 adsorption to epithelial cells. For that purpose, peptides conjugates were added to epithelial cells together with HSV-1 and were present only during the period of infection at 4°C (Figure 2C). We found that the viral yield and titer were significantly reduced by DAL-PEG-LK6 and CPP1-PEG-LK6. In the presence of DAL-PEG-DK5, we noticed a decrease in viral yield of up to ∼86% in qPCR (Figure 2D) and ∼91% using titration assay (Figure 2E). In the case of CPP1-PEG-LK6, we observed a decreased in virus replication reaching ∼95% using qPCR (Figure 2D) and ∼97% in the titration assay (Figure 2E). To confirm the above data, we applied confocal imaging, showing that both peptide conjugates inhibit virus attachment to the cell surface, which can explain the reduced replication of the pathogen (Figure 2F). Moreover, we confirmed the higher antiviral efficiency of CPP1-PEG-DK5 in contrast to DAL-PEG-LK6 samples (Figures 2F,G). Collectively, the obtained data show that DAL-PEG-LK6 and CPP1-PEG-LK6 exert a significant antiviral effect by blocking the interaction between the virus and the host cell.

### 3.3 CPP1-PEG-LK6 binds to heparan sulfate

**Figure 3:**
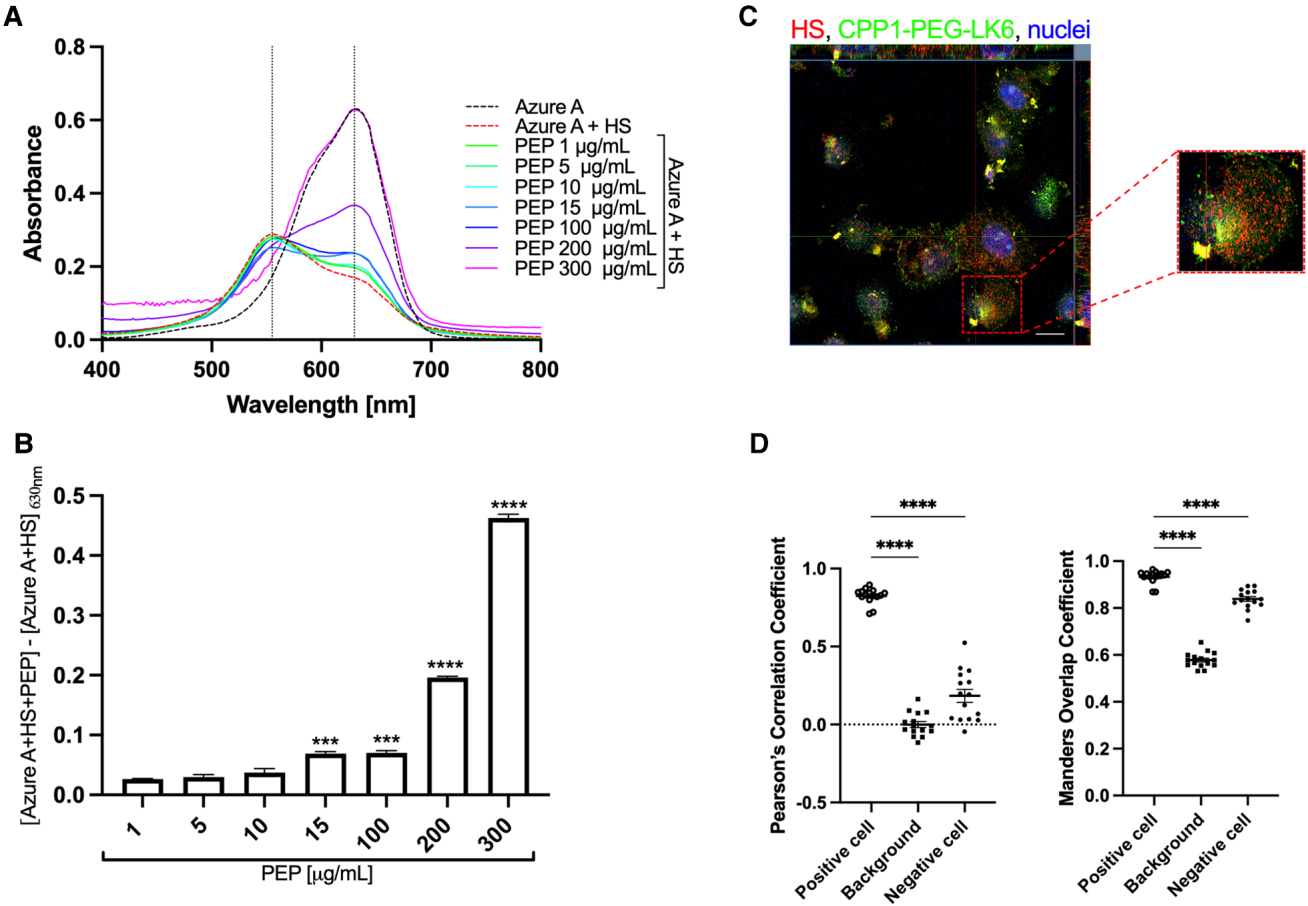
CPP1-PEG-LK6 peptide conjugate binds to heparan sulfate. (**A**) Absorption spectra of Azure A (23 μg/ml) in the presence of HS (0.2 mg/ml) and different concentrations of CPP1-PEG-LK6 (PEP, 1–300 μg/ml). (**B**) The concentration dependence of CPP1-PEG-LK6 from subtraction of the AzureA + HS absorbance from the absorption of AzureA + HS + PEP measured at 630 nm. ***P < 0.001; ****P < 0.0001; one-way ANOVA. (**C**) The interaction of CFS labeled CPP1-PEG-LK6 with HS was confirmed by confocal microscopy (HS, red; CPP-PEG-LK6, green; nuclei, blue). Scale bar: 10 μm. (**D**) Pearson's correlation coefficients (PCCs) and Manders overlap coefficient (MOC) tests were run to quantify the colocalization degree. PCCs values can range from −1 (an anti-colocalization), to 0 (no-colocalization), or +1 (maximal colocalization). MOC measurement ranges from 0 to +1. Data are presented as the average ± SEM. ****P < 0.0001; one-way ANOVA.

To examine in detail the role of tested conjugates in the inhibition of HSV-1 interaction with the host cells, we focused on the heparan sulfate proteoglycans (HSPGs), which are described as crucial cell membrane components that recognize HSV-1 glycoproteins gB and/or gC (44). We selected the CPP1-PEG-LK6 conjugate as the most efficient antiviral compound and determined its interaction with heparan sulfate (HS) using Azure A metachromatic assay. We found that the CPP1-PEG-LK6 conjugate releases Azure A from HS, manifested by an increase in the peak at 630 nm (Figures 3A,B). The obtained data indicate that HS is complexed with CPP1-PEG-LK6, and the effect increases with peptide concentration (Figure 3B). Importantly, the non-toxic concentration of peptide (15 μg/ml) used during *in vitro* experiments displayed a significant increase in absorbance at 630 nm. To confirm the interaction of CPP1-PEG-LK6 with heparan sulfate proteoglycans anchored to the cell membrane, we applied confocal microscopy imaging (Figure 3C). We observed colocalization between HS (red) on the cell surface and the peptide conjugate (green). The degree of colocalization was 0.82 ± 0.02 and 0.93 ± 0.01, as quantified using Pearson's correlation and Manders overlap coefficient, respectively (Figure 3D). Collectively, these data indicate that CPP1-PEG-LK6 inhibits virus replication, most likely by interacting with HS molecules on the host cell surface.

### 3.4 CPP1-PEG-LK6 inhibits Acyclovir-resistant HSV-1 clinical strains

**Figure 4:**
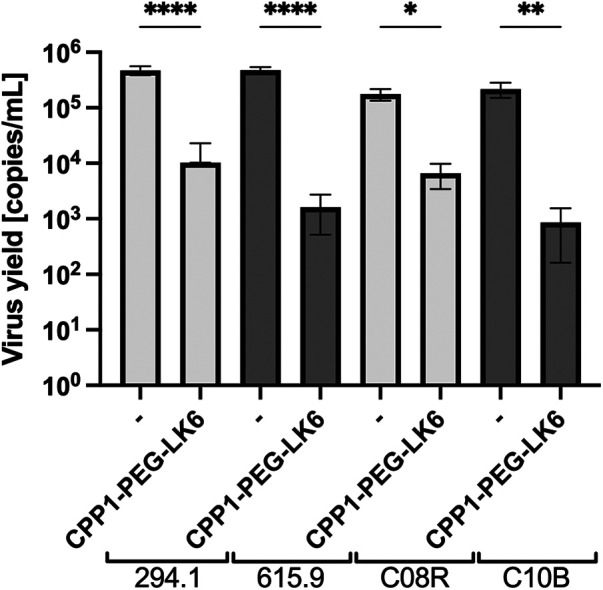
Viral replication assay was performed for two sets of clinical strains. Cells were pre-treated with DAL-PEG-LK6 and CPP1-PEG-LK6 (15 μg/ml) and infected with clinical strains in the presence of tested conjugates and cultivated in their presence. 48 h p. i. cell culture supernatants were collected, and viral yield was quantified by qPCR. Data are presented as the average ± SD. Similar results were obtained in at least duplicates in three independent experiments. *P < 0.1, **P < 0.01, ****P < 0.0001; one-way ANOVA.

On the basis of the obtained results, CPP1-PEG-LK6 has emerged as a promising antiviral compound against HSV-1. Thus, we compared its activity to acyclovir, the most common and widely available antiviral drug. As shown in Table 3, acyclovir displays higher potency against HSV-1 with an IC_50_ of 0.138 μg/ml (SI 724.63) compared to CPP1-PEG-LK6. Next, to assess whether CPP1-PEG-LK6 displays antiviral effects against HSV-1 clinical strains, a viral inhibition assay was performed. To verify the potential usefulness of the peptide conjugate for antiviral therapy, we tested anti-herpetic activity of CPP1-PEG-LK6 against two sets of strains. One set is 294.1 (pre-treatment) and 615.9 (resistant to acyclovir) derived from a patient with acyclovir-resistant esophagitis. The second set is C08R (pre-treatment), and C10B (resistant to acyclovir and foscarnet) from an immunocompromised patient with recurrent herpes keratitis that failed to respond to acyclovir (45, 46). The results (Figure 4) show that our compound inhibits all selected HSV-1 clinical strains regardless of their sensitivity to acyclovir or foscarnet.

**Table 3:** CPP1-PEG-LK6 and Acyclovir antiviral activity against HSV-1.

Compound	CC_50_^a^ [μg/ml]	IC_50_^b^ [μg/ml]	SI^b^
Acyclovir	>100	0.138	724.63
CPP1-PEG-LK6	23.40	0.942	24.84

^a^ The IC_50_ (inhibitory concentration that reduced viral replication by 50%) and CC_50_ (50% cytotoxic concentration) are expressed as the mean [μg/ml] of two independent experiments.

^b^ SI, selectivity index, determined by the ratio of CC_50_ to IC_50_.

### 3.5 Antiviral activity of CPP1-PEG-LK6 in OTG model

**Figure 5:**
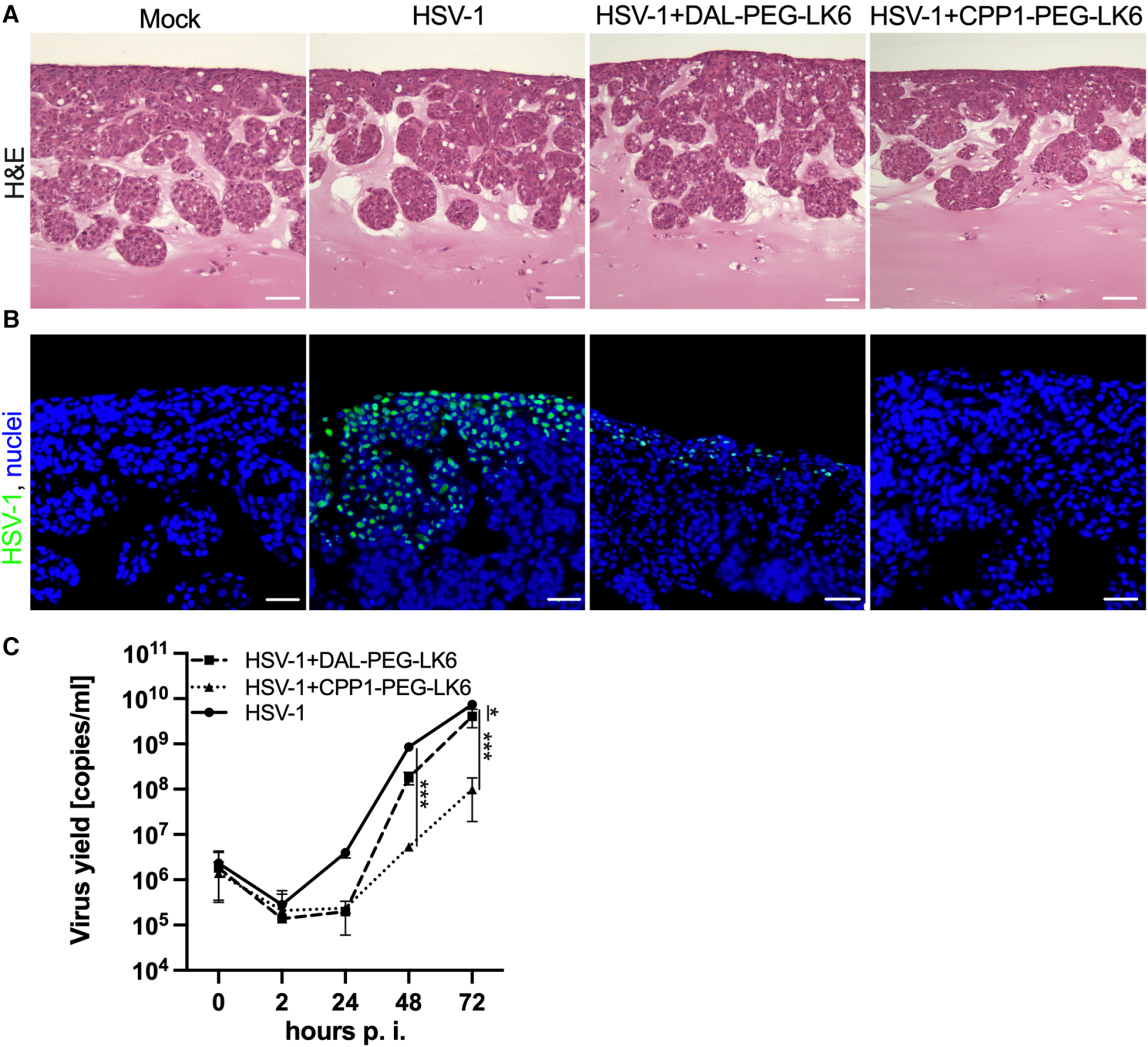
Antiviral activity of temporin-1CEb analogues in 3D OTG model. (**A**) H&E stained sections of OTG model infected with HSV-1 in the absence or presence of DAL-PEG-LK6 and CPP1-PEG-LK6 (15 μg/ml). Scale bar: 50 μm. (**B**) Immunofluorescence staining with a HSV-1 antibodies was performed to evaluate infection of the OTG in the presence of both tested compounds (virus, green; nuclei, blue). Scale bar: 50 μm. (**C**) Replication kinetics of HSV-1 in the presence of both peptide conjugates was analyzed. Apical washes of infected OTG were collected over time until 72 h p.i., and the number of viral genomic DNA copies was determined by qPCR. Results are presented as an average ± SD of two independent experiments in duplicates. *P < 0.01, ***P < 0.001; 2way ANOVA.

To evaluate whether the tested conjugate is effective in tissue, we applied a 3D OTG model generated by TIGKs cells and gingival fibroblasts cultivated in an air-liquid interface (34). In Figure 5A, we present sections of OTG infected with HSV-1 in the presence of DAL-PEG-DK5 and CPP1-PEG-LK6. H&E staining revealed no changes in the morphology of epithelial cells after infection nor peptides stimulation. Furthermore, the effect of both peptides conjugates during consecutive days following HSV-1 infection was examined. Apical washes of infected OTG were collected over time until 72 h post-infection for DNA isolation, and the number of viral genomic DNA copies was analyzed by qPCR to determine the level of HSV-1 infection. As shown in Figure 5C, infection in the presence of CPP1-PEG-LK6 markedly inhibited HSV-1 replication. This compound reduced viral yields by 3 and 2 logs at 48 and 72 h post-viral inoculation, respectively. DAL-PEG-LK6 showed inhibition of viral infection (1 log) only 24 h post-infection. These results were confirmed by immunohistological detection of viral infection (Figure 5B). HSV-1-positive cells were observed only in the top layer of OTG in virus-infected sections, while they were absent in the presence of CPP1-PEG-LK6 and in mock infected-cultures. On the contrary, HSV-1-positive cells could be detected in sections with cells infected in the presence of DAL-PEG-DK5. These data showed that the tested conjugates efficiently inhibit the viral infection in tissue.

## 4 Discussion

HSV infections are among the most prevalent human diseases globally (1). These infections are a common problem in patients with immunosuppression, those undergoing chemotherapy, and others suffering from chronic inflammation of the oral mucosa and gums (47–51). It could lead to life-threatening systemic HSV infections mainly occur in individuals with compromised immune system experiencing concurrent virus reactivation (52). Moreover, it has been shown that HSV-1 is present in the elderly human brain in a latent form and reactivates periodically, causing harmful effects on cognition and eventually development of Alzheimer's disease (53). For several decades, acyclovir has been the primary treatment for herpes infections. Nevertheless, prolonged antiviral therapy, particularly in chronically ill patients, has led to the emergence of resistance to acyclovir and other acyclic guanosine analogues, including valacyclovir or penciclovir (2, 47). Therefore, there is a clear medical need for the development of new effective antivirals that offer good bioavailability and tolerability with minimal side effects. This study reports on the anti-herpetic activity of a peptide conjugate consisting of a cell-penetrating peptide and a temporin-1CEb analogue—CPP1-PEG-LK6.

Temporins attract the attention of the scientific world as potent antiviral compounds with a limited possibility of developing virus tolerance (29, 30, 54). The majority of them have been found to partially affect early stages of the HSV-1 life cycle with clearly defined molecular mechanism of activity. Among them are temporins (B, G, Sha and L) that exhibit virucidal activity against HSV-1 by directly interacting with viral glycoproteins gC and gB (29–31, 55). It has been shown that the inhibitory activity of temporins B and G is not attributed to interference with cellular receptors (29, 31). In this respect, the studied analogue of temporin-1CEb has a different molecular target, interacting with HS, the negatively charged glycosaminoglycans on the cell surface, which is considered one of the receptors recognizing HSV-1 (56). This interaction is facilitated by positively charged domains on viral glycoproteins gC and gB (56, 57). The attachment enables the virus to surf towards the cell body, making entry receptors available (57). Subsequently, gD binds to one of several host cell entry receptors, such as nectins, herpesvirus entry mediator (HVEM), or 3-O-sulfated heparan sulfate (3-OS HS), leading to conformational changes that facilitate the recruitment of gB, gH and gL (58–61). The complex formed between glycoproteins and cellular receptors results in the merging of the lipid bilayer with the virus, allowing penetration of the viral particle into the host cytoplasm (59). The presented inhibition of pathogen attachment to host cells and neutralizing HSV-1 entry by the tested conjugate suggests its effectiveness in the early stages of viral infection. In this regard the temporin-1CEb analogue is similar to another amphibian AMPs, dermaseptin, which exhibits anti-herpetic activity against HSV-1 and HSV-2 by interacting with carboxyl groups in HS (62, 63). Apart from limiting the entry of the virus into the cell, we cannot exclude another mechanism of action, as the most efficient antiviral conjugate was composed of a cell-penetrating peptide. We can assume that the tested conjugate additionally acts on the intracellular stages of virus development, but this requires further research.

Apart from having a different molecular mechanism for limiting HSV-1 infection, the tested temporin also differs in efficiency. Significantly lower IC_50_ was noticed for temporin-1CEb (0.942 μg/ml) compared to temporin B (93 μg/ml). However, it should be underlined that the mentioned temporins were tested in different models of epithelial cells. In our studies, we initially utilized the Vero E6 cell line, which has been a long-standing choice in herpesviruses research (40, 41). Subsequently, we used the TIGKs cell model to demonstrate that the observed inhibition of viral attachment and replication is not cell-type dependent. Finally, we confirmed the antiviral effect using a 3D model that resembles human gingiva (34). Notably, in all three applied cell models, CPP1-PEG-LK6 demonstrated a comparable and significant antiviral effect.

The observation indicating the sensitivity of clinical strains is a promising result. We investigated two sets of strains, which includes a pre-treatment drug-sensitive (294.1 and C08R) and drug-resistant viruses (615.9 and C10B) (45, 46), observing the inhibition of replication (by ∼3 logs) in all tested strains by CPP1-PEG-LK6, regardless of their sensitivity to acyclovir or foscarnet. These data highlights the significant potential application of our molecule in treating immunocompromised patients with recurrent herpes infections.

Overall, our data demonstrate for the first time the ability of temporin-1CEb analogue combined with a cell-penetrating peptide to interfere with the replication of HSV-1. Many questions remain open, for example, whether the peptide will be effective against latent forms of HSV-1 or whether it will also affect HSV-2. Nonetheless, the data presented herein indicate that the CPP1-PEG-LK6 conjugate is a promising candidate for the treatment of mucocutaneous infections caused by HSV-1, including resistant strains, thus offering perspectives for the development of new antiviral therapeutics.

## Data Availability

The original contributions presented in the study are included in the article/**Supplementary Material**, further inquiries can be directed to the corresponding authors.
