# Supplementary material for: Antiviral activity of temporin-1CEb analogues against gingival infection with herpes simplex virus type 1
Source: Front Oral Health. 2024 Jun 17;5:1430077. doi: 10.3389/froh.2024.1430077 (PMC11215077; doi:10.3389/froh.2024.1430077)
Supplement: Supplementary file 1 [file Datasheet1.docx]

Supplementary Material

Antiviral activity of temporin-1CEb analogues against gingival infection with herpes simplex virus type 1

Anna Golda^1*^, Paulina Kosikowska-Adamus^2^, Marta Wadowska^1^, Ewelina Dobosz^1^, Jan Potempa^3^, Joanna Koziel^1*^

*** Correspondence:** Joanna Koziel: [joanna.koziel@uj.edu.pl](mailto:joanna.koziel@uj.edu.pl), Anna Golda: [anna.b.golda@uj.edu.pl](mailto:anna.b.golda@uj.edu.pl)

# Supplementary Figures


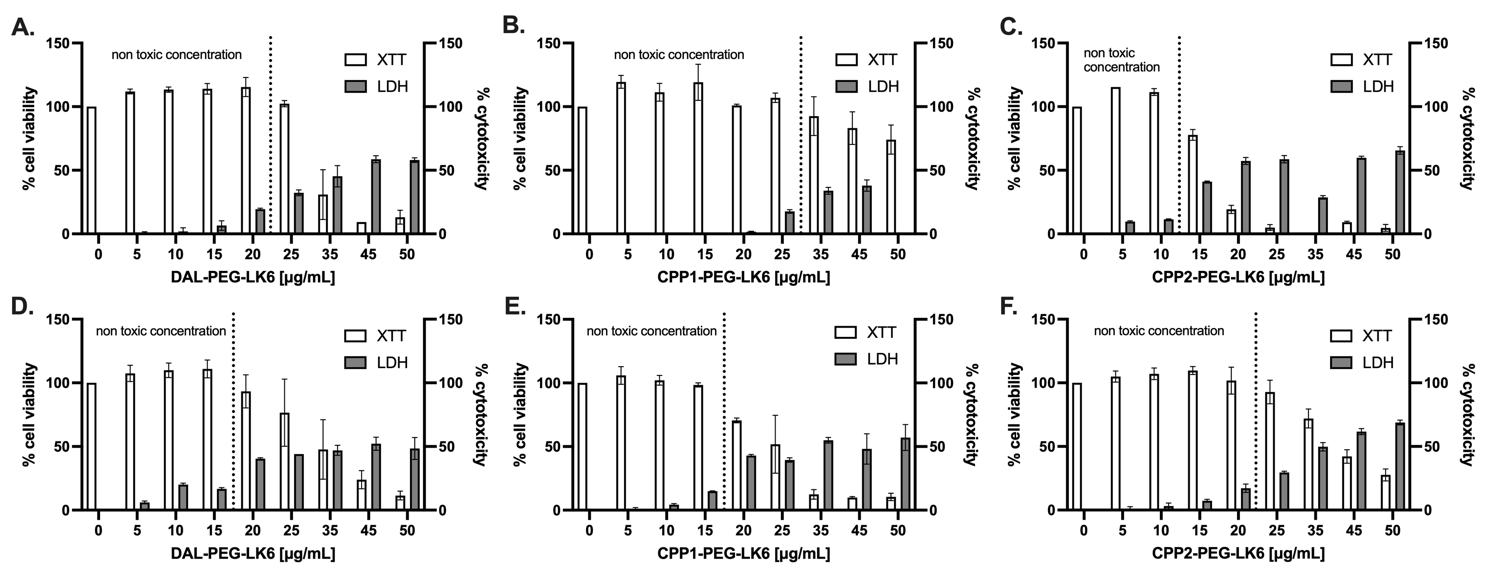


**Supplementary Figure 1.** Cell viability and cytotoxicity of temporin-1CEb analogues were determined by XTT (open bars) and LDH (grey bars) test, respectively. The influence of DAL-PEG-LK6, CPP1-PEG-LK6, CPP2-PEG-LK6 on physiology of Vero E6 (**A, B, C**) and TIGKs (**D, E, F**) was tested in the range of 5−50 μg/ mL concentrations. Cell viability and cytotoxicity was calculated in reference to untreated cells. Results are presented as an average ± SD of three independent experiments in duplicates.


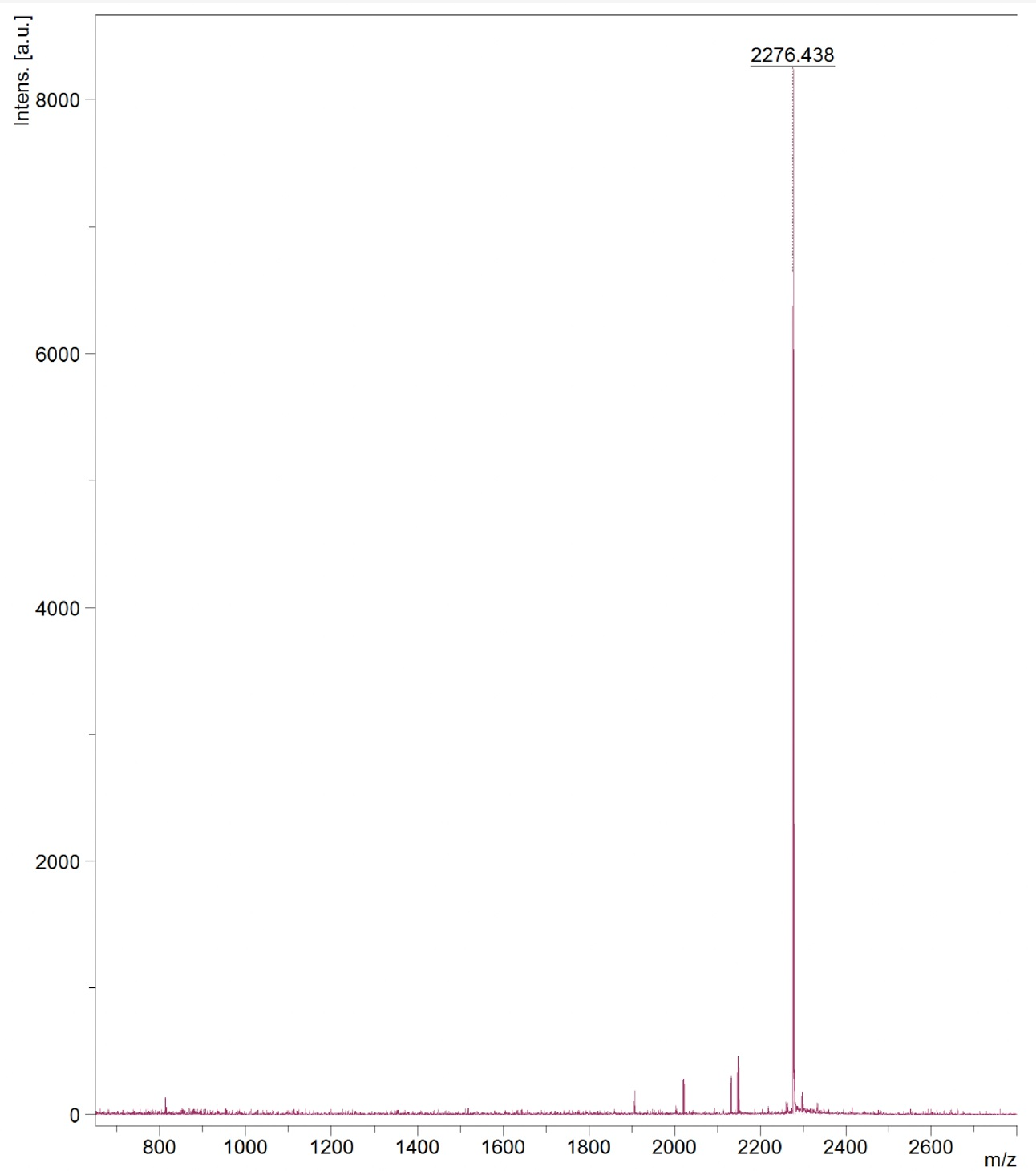

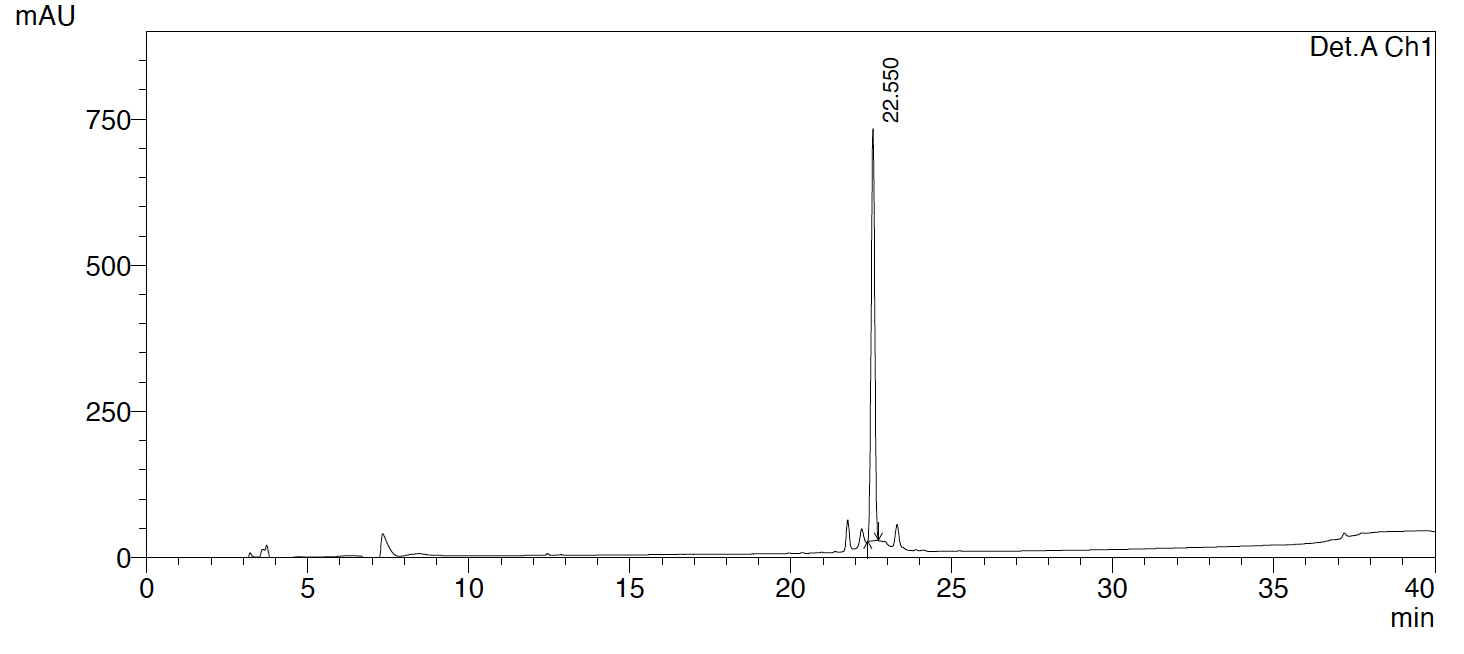


**Supplementary Figure 2.** The mass spectrometry analysis (Biflex III MALDI TOF with CCA/DHB matricies) and RP-HPLC chromatograms (Shimadzu HPLC system with Phenomenex Jupiter 4μ Proteo 90 Å column, 250 × 4.60 mm column) obtained for DAL-PEG-DK5 peptide.


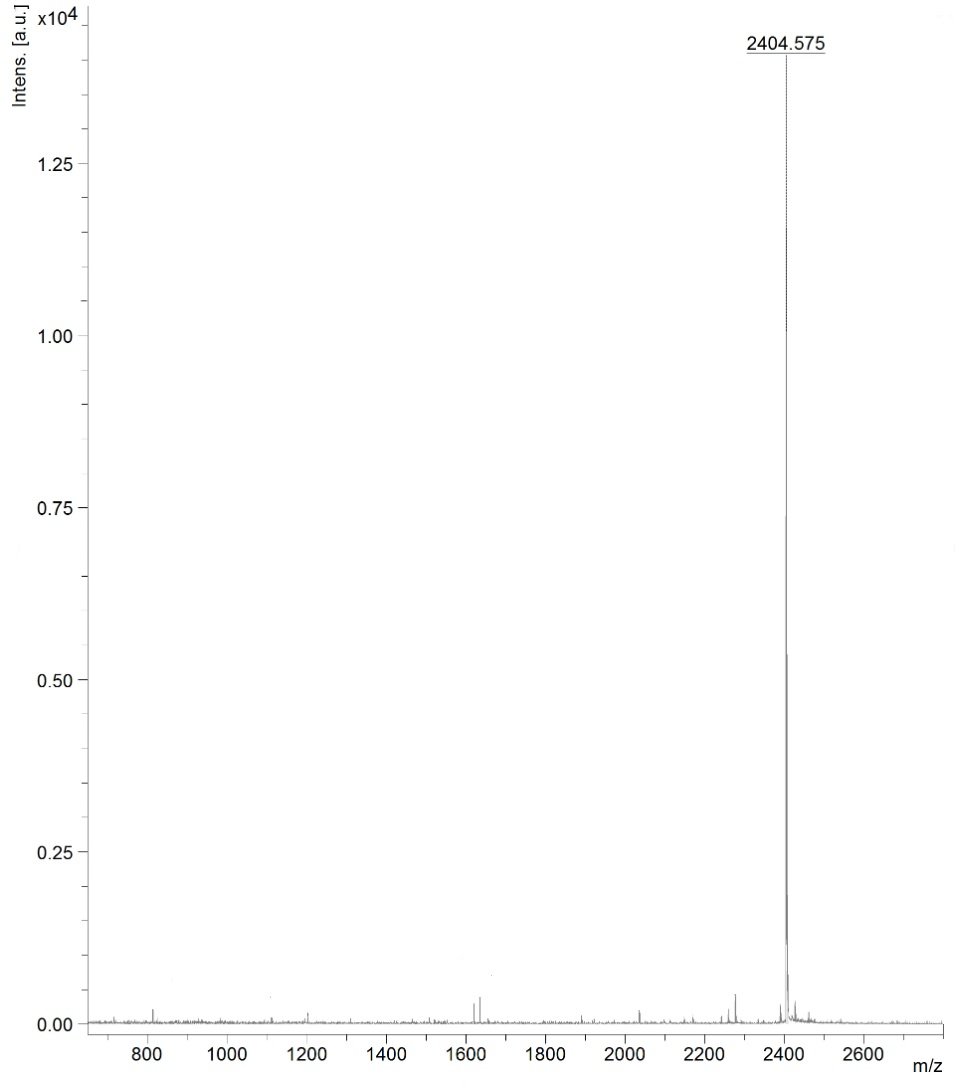


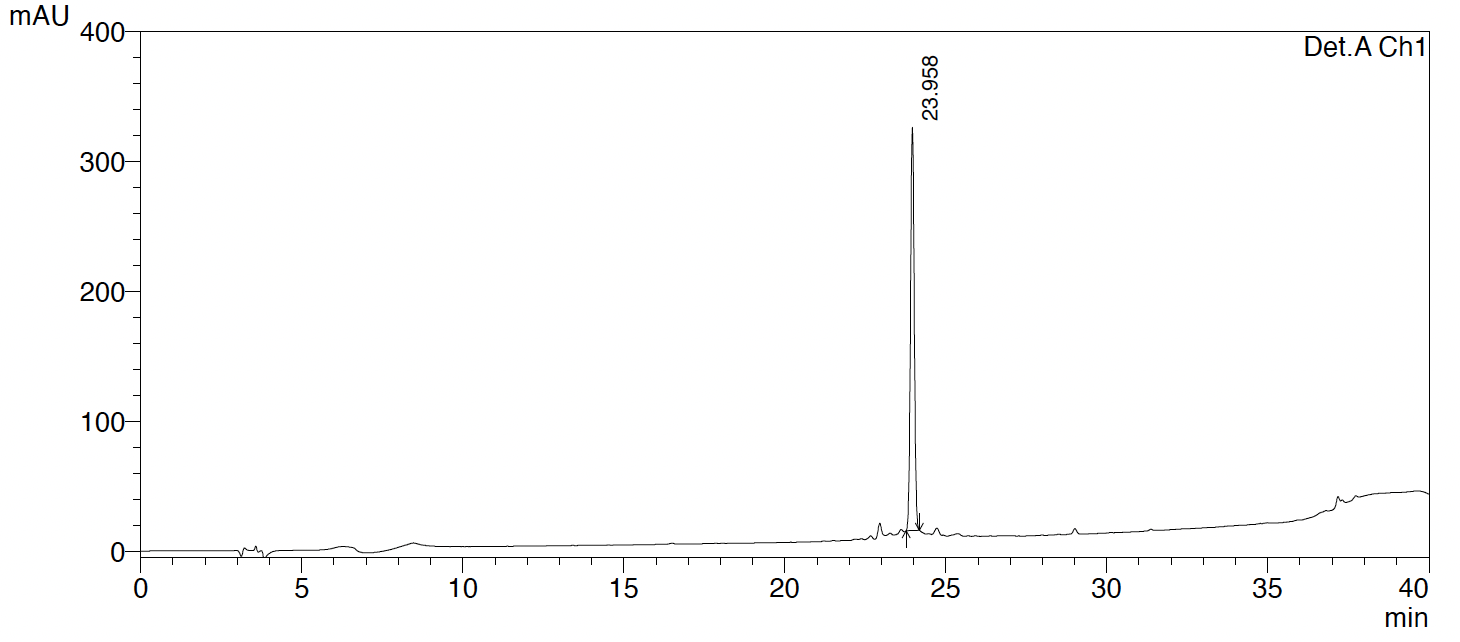


**Supplementary Figure 3.** The mass spectrometry analysis (Biflex III MALDI TOF with CCA/DHB matricies) and RP-HPLC chromatograms (Shimadzu HPLC system with Phenomenex Jupiter 4μ Proteo 90 Å column, 250 × 4.60 mm column) obtained for DAL-PEG-LK6 peptide.


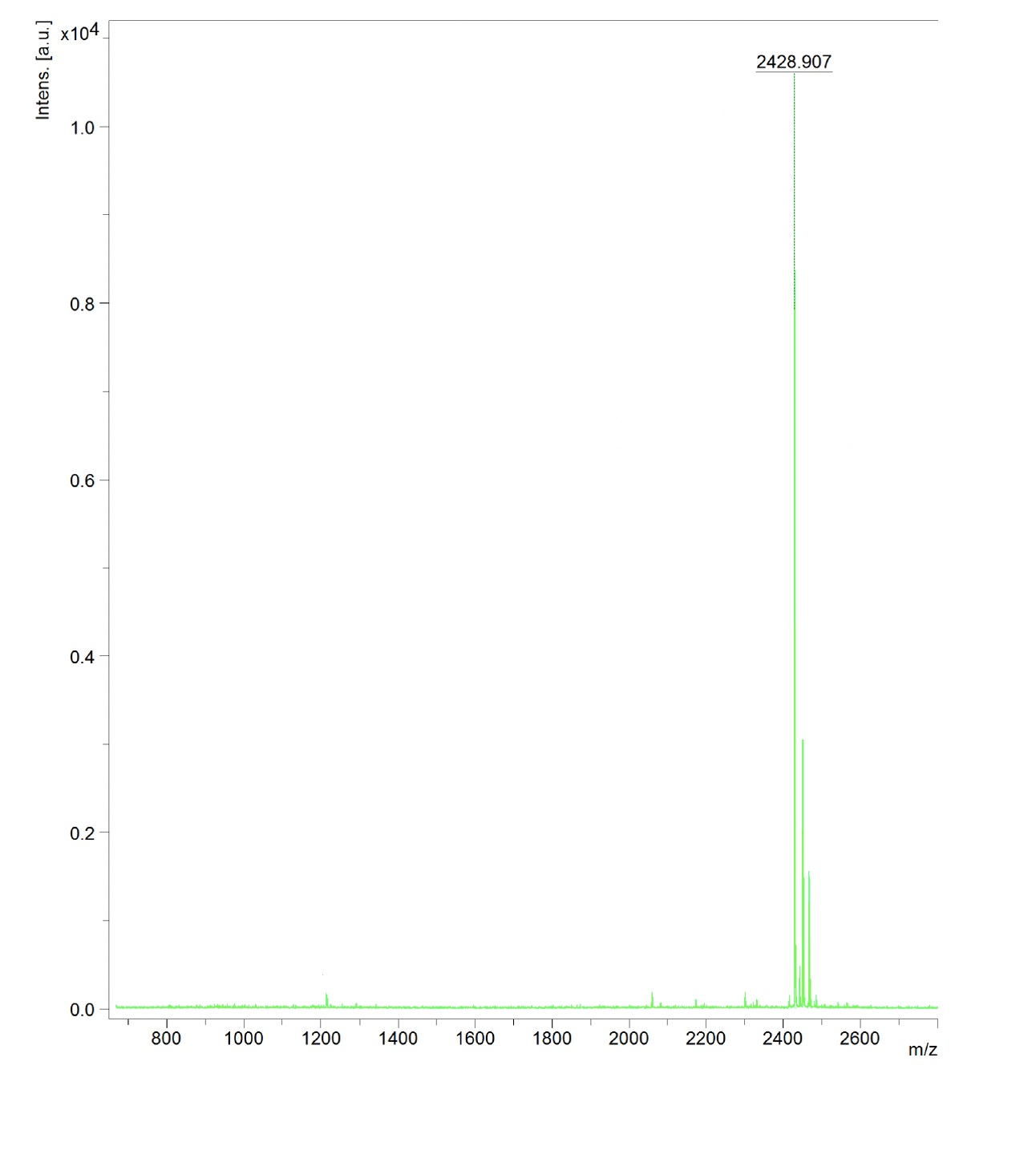


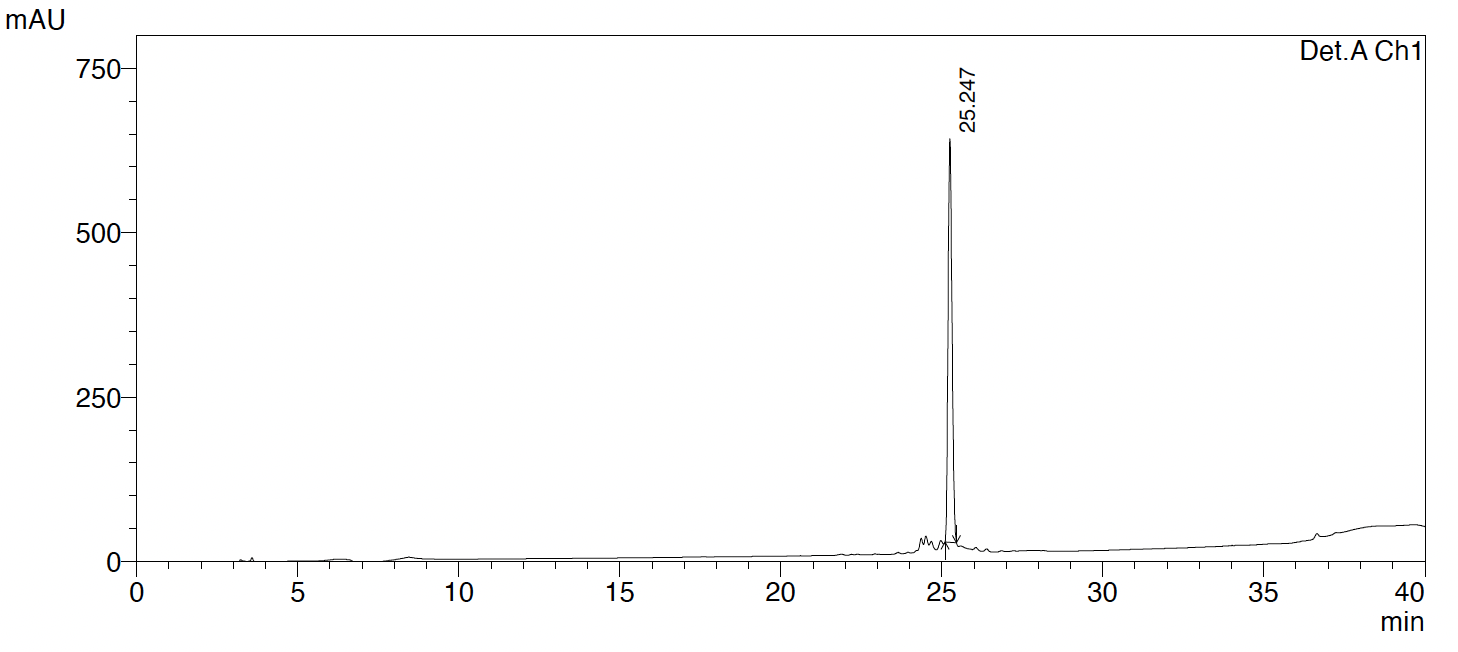


**Supplementary Figure 4.** The mass spectrometry analysis (Biflex III MALDI TOF with CCA/DHB matricies) and RP-HPLC chromatograms (Shimadzu HPLC system with Phenomenex Jupiter 4μ Proteo 90 Å column, 250 × 4.60 mm column) obtained for CPP1-PEG-LK6 peptide.


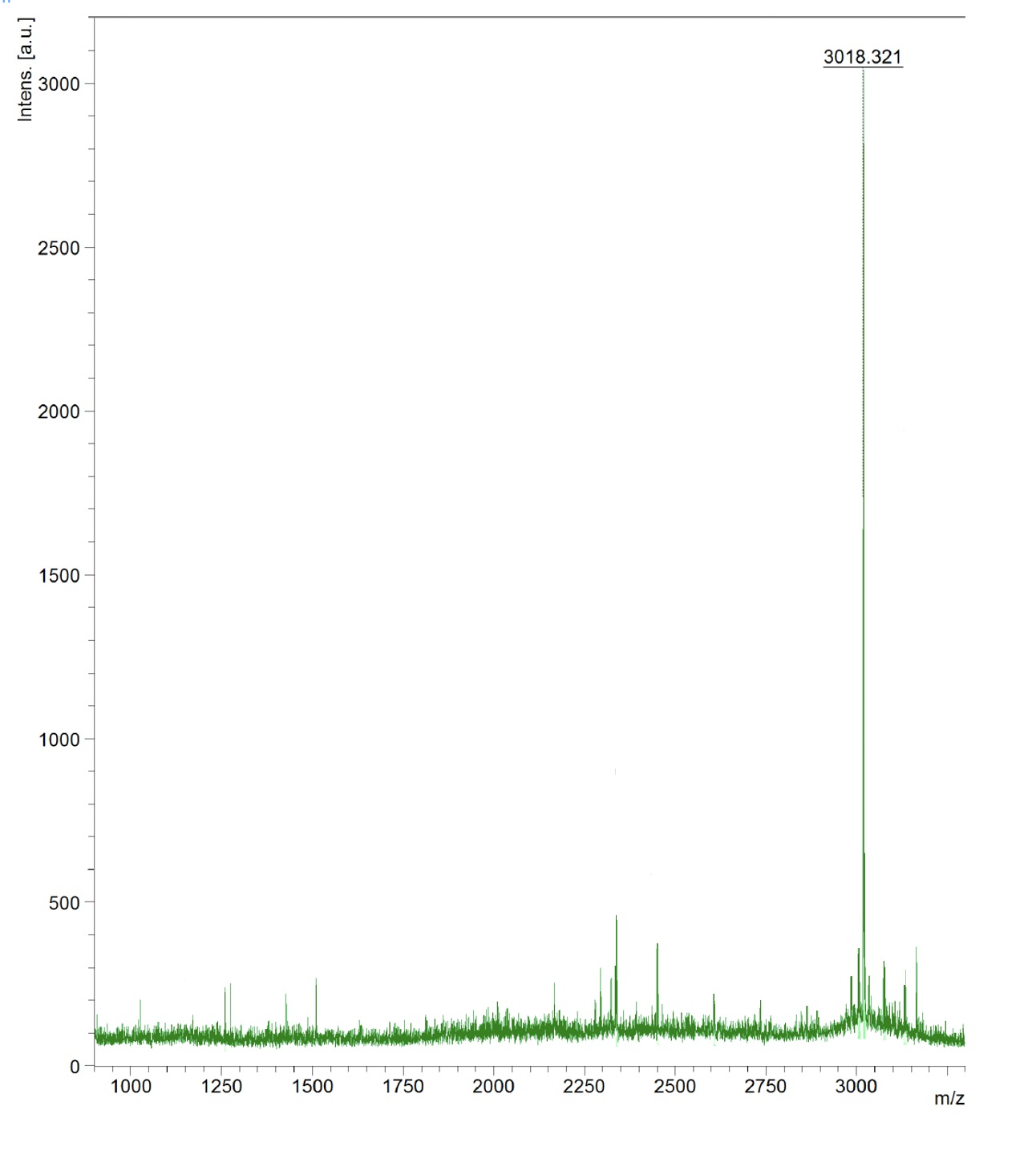


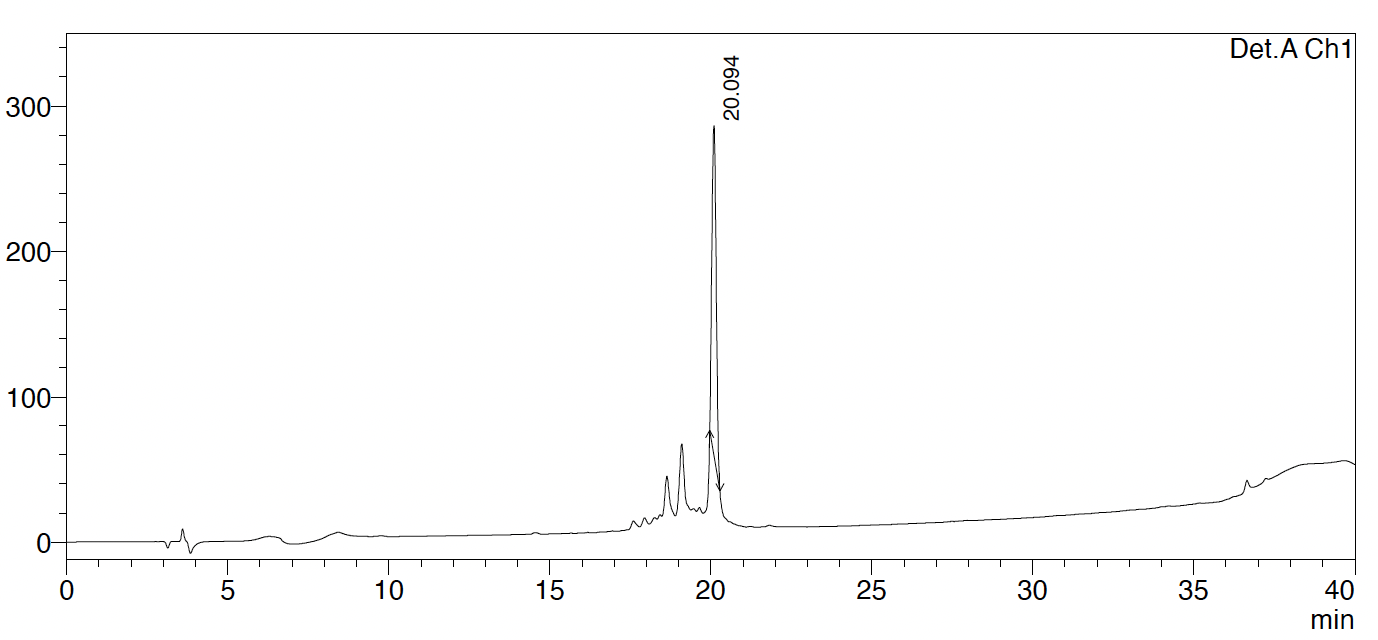


**Supplementary Figure 5.** The mass spectrometry analysis (Biflex III MALDI TOF with CCA/DHB matricies) and RP-HPLC chromatograms (Shimadzu HPLC system with Phenomenex Jupiter 4μ Proteo 90 Å column, 250 × 4.60 mm column) obtained for CPP2-PEG-LK6 peptide.
